# Cardiovascular protection effect of chlorogenic acid: focus on the molecular mechanism

**DOI:** 10.12688/f1000research.26236.1

**Published:** 2020-12-15

**Authors:** Mifetika Lukitasari, Mohammad Saifur Rohman, Dwi Adi Nugroho, Nashi Widodo, Nur Ida Panca Nugrahini

**Affiliations:** 1 Department of Nursing, Faculty of Medicine, Brawijaya University, Malang, East java, +62, Indonesia; 2 Department of Cardiology and Vascular Medicine, Faculty of Medicine, Brawijaya University-Saiful Anwar General Hospital, Malang, East java, +62, Indonesia; 3 Department of Herbal Medicine, Cardiovascular research group, Faculty of Medicine, Brawijaya University, Malang, East java, +62, Indonesia; 4 Department of Biology, Faculty of Mathematics and Natural Science, Brawijaya University, Malang, East java, +62, Indonesia; 5 Department Agricultural Product Technology, Brawijaya University, Malang, East java, +62, Indonesia

**Keywords:** Chlorogenic acid, polyphenol, endothelial dysfunction, vascular health

## Abstract

Vascular endothelial cells have a variety of functions such as the control of blood coagulation, vascular permeability, and tone regulation, as well as quiesce of immune cells. Endothelial dysfunction is a cardiovascular events predictor, which is considered the initial stage in atherosclerosis development. It is characterized by alterations in endothelium functions due to imbalanced vasodilators and vasoconstrictors, procoagulant and anticoagulant mediators, as well as growth inhibitor and promotor substances. Chlorogenic acid (CGA) is the primary polyphenol in coffee and some fruits. It has many health-promoting properties, especially in the cardiovascular system. Many studies investigated the efficacy and mechanism of this compound in vascular health. CGA has several vascular benefits such as anti-atherosclerosis, anti-thrombosis, and anti-hypertensive. This review focuses on the molecular mechanism of CGA in vascular health.

## Introduction

Vascular endothelial cells have a variety of functions, such as the control of blood coagulation, vascular permeability, and tone regulation, as well as quiesce of immune cells ^1^. Endothelial dysfunction (ED) is considered as a cardiovascular events predictor, and it is characterized by alterations in endothelium functions that tend to be vasoconstricted, procoagulant, and prothrombotic ^2^. Chlorogenic acid (CGA) is a compound of phenol that consists of a caffeic and quinic acid moiety; therefore, it is also called 5-O-caffeoylquinic acid (5-CQA), although many authors refer to it as 3-CQA. A cup of coffee (200 ml) consists of 20–350 mg CGA, which contains 35–175 mg of caffeic acid. Therefore, an average coffee drinker consumes 0.5–1 g of CGA daily ^3^. Moreover, this compound is found in fruits, such as pears, strawberries, eggplant, apples, blueberries, and tomatoes ^4^. CGA is widely studied because of its health properties, such as anticancer, antineurodegenerative, antidiabetic, anti-inflammatory, antilipidemic, and antioxidant. This review discusses CGA’s effects on vascular health, focussing on its molecular mechanism.

## Endothelial dysfunction (ED)

The functions of vascular endothelial cells includes vascular permeability, and tone regulation, as well as quiesce of immune cells. Vascular tone is mainly regulated by nitric oxide (NO). Healthy endothelium are protected from adhesion and aggregation through the release of NO, prostacyclin, and platelet ADP degradation ^5^. As long as the endothelial layer is healthy and intact, platelets in circulation remain in an inactive state. ED is a cardiovascular events predictor and considered as the initial stage of atherosclerosis development. It is characterized by alterations in endothelium functions due to imbalanced vasodilators and vasoconstrictors, procoagulant and anticoagulant mediators, as well as growth inhibitor and promotor substances ^6^.

Adiponectin is the biomarker of some cardiovascular disease risk factors such as diabetes, metabolic syndrome, atherosclerosis, or obesity. This adipokine has antioxidant, insulin-sensitizing, and anti-inflammatory properties ^7^. Both of its receptors, AdipoR2 and AdipoR1 have anti-atherogenic activity through the improvement of PPAR and AMPK ligand activity ^8^. In endothelial cells, this substance may downregulate adhesion molecules expression such as ICAM-1, which facilitates monocyte attachment to the endothelium by inhibiting TNF- *α*-mediated activation of NF- *κ*B. The activity of endothelial nitric oxide synthase (eNOS) can also be increased by adiponectin by facilitating its phosphorylation at Ser1177 via AMPK. It also inhibits reactive oxygen species (ROS) production by oxidized low-density lipoprotein (oxLDL) in cultured endothelial cells. These effects show that high adiponectin levels may prevent atherosclerosis ^9– 11^.

## Chlorogenic acid (CGA)

CGA is a compound of phenol that consists of caffeic and quinic acid moiety. It is also called 5-O-caffeoylquinic acid (5-CQA), although some authors refer to it as 3-CQA. This compound is the primary polyphenol in coffee. A cup of coffee (200 ml) consists of 20–350 mg CGA, which contains 35–175 mg of caffeic acid ^12^. Therefore, an average coffee drinker consumes 0.5–1g of CGA daily. In addition, this compound is found in some fruits, such as pears, blueberries, eggplant, strawberries, apples, and tomatoes. It is widely studied since it has several healthy properties, such as antioxidant, anti-inflammatory, anticancer, antilipidemic, antidiabetic, anti-hypertensive, and anti-neurodegenerative.

## Mechanism of CGA in inhibiting atherosclerosis

Atherosclerosis is a multifactorial inflammatory disease initiated by oxidative stress and foam cell formation. Foam cell formation can be inhibited by inducing cholesterol efflux to lipid poor apoplipoprotein such as ApoA1. ABCG1 and ABCA1 are cholesterol transporters that play a significant role in mediating cholesterol efflux to high density lipoprotein. These molecules are regulated by nuclear transcriptional factors LXRa and PPARc ^13^. CGA has been shown to significantly increase mRNA levels of PPARγ, LXRα, ABCA1 and ABCG1, as well as the transcriptional activity of PPARγ. In addition, a cholesterol efflux assay showed that three major metabolites, caffeic, ferulic and gallic acids, significantly stimulated cholesterol efflux from RAW264.7 cells. These results suggest that CGA potently reduces atherosclerosis development in ApoE ^−/−^ mice and promotes cholesterol efflux from RAW264.7 macrophages ^14^.

CGA also has a dual PPAR α/γ agonist. Previous studies revealed that its administration enhanced AMPK phosphorylation, adiponectin, and its receptors ^15^. These mechanisms indirectly have a beneficial effect on preventing ED, and AMPK activation has been shown to inhibit protein kinase C as a potent atherogenic substance ^16^. Several studies have revealed the effect of PPARγ agonists on improving ED. PPARγ agonist reverses oxLDL-induced ED through AMPK activation, which consequently enhances eNOS activity. Also, it increased adiponectin levels as a potent anti-inflammatory agent ^17– 19^. CGA and its major metabolite, caffeic acid, have antioxidant effects *in vitro* that alter LDL oxidation. The antioxidant effect of this compound increases LDL resistance to *ex vivo* oxidation ^14^.

Lysophosphatidylcholine (LPC) is the primary atherogenic compound of oxLDL. It increases intracellular calcium through store-operated channels (SOCs). Moreover, it decreases cell viability and increases ROS generation. The expression of transient receptor potential canonical (TRPC) channel is significantly increased by LPC treatment ^20, 21^. Previous studies showed that CGA inhibited ROS production by reducing TRPC1 expression, and therefore restored cell viability. Meanwhile, it inhibits LPC-induced Ca ^2+^ influx through SOC. Thus, CGA protects endothelial cells from LPC injury and consequently inhibits atherosclerosis ^22^.

Hemeoxygenase-1 is induced in response to ROS in endothelial cells, which plays a role in preventing damage. CGA reduces xanthine oxidase-1 and ROS, as well as enhances hemeoxygenase-1 and superoxide dismutase levels in endothelial cells. Its effects were described on endothelial function in an isolated aortic ring from mice. It was also shown in this study to decrease HOCl-induced oxidative damage in endothelial cells, and this mechanism is related to the induction of hemeoxygenase-1 and NO production ^23^. Consuming coffee high in CGA repairs ED by reducing oxidative stress. Previous studies showed that oxidative stress played an essential role in ED ^24^. However, CGA can prevent this, owing to its antioxidant activity. Also, it inhibits vascular and intercellular adhesion molecule-1, as well as the expression of monocyte chemotactic protein-1 ^25^. In addition, it prevents T2DM and blocks α-glucosidase activity. It was also reported that CGA inhibits disorder of the endothelium through this activity ^26^.

## Mechanism of CGA in inhibiting platelet activation

Hypertension, diabetes and dyslipidaemia, well-known cardiovascular-event risk factors, augment inflammation and might induce platelet adherence to the endothelial layer even in the absence of an activator or injury. Meanwhile, damaged endothelium triggers the release of collagen and von Willebrand factor (vWF) from the extracellular matrix and some derivatives such as thrombin, ADP, and thromboxane A2 (TXA2) that finally lead to platelet activation ^27^. Platelet biomarkers are elevated in risk factors of cardiovascular disease such as hypertension, diabetes mellitus, and obesity ^28^. In atherosclerosis and thrombosis, an elevated level of P-selectin becomes the predictive biomarker of potential adverse cardiovascular events like stroke and myocardial infarction ^29^. P-selectin glycoprotein ligand-1 (PSGL-1) plays a crucial role in inflammation and the initial adhesion of leukocytes to areas of injury. Furthermore, it plays an essential role in thrombosis and homeostasis through PSGL-1 signalling and GPIbα in platelets ^30^.

**Figure 1.**
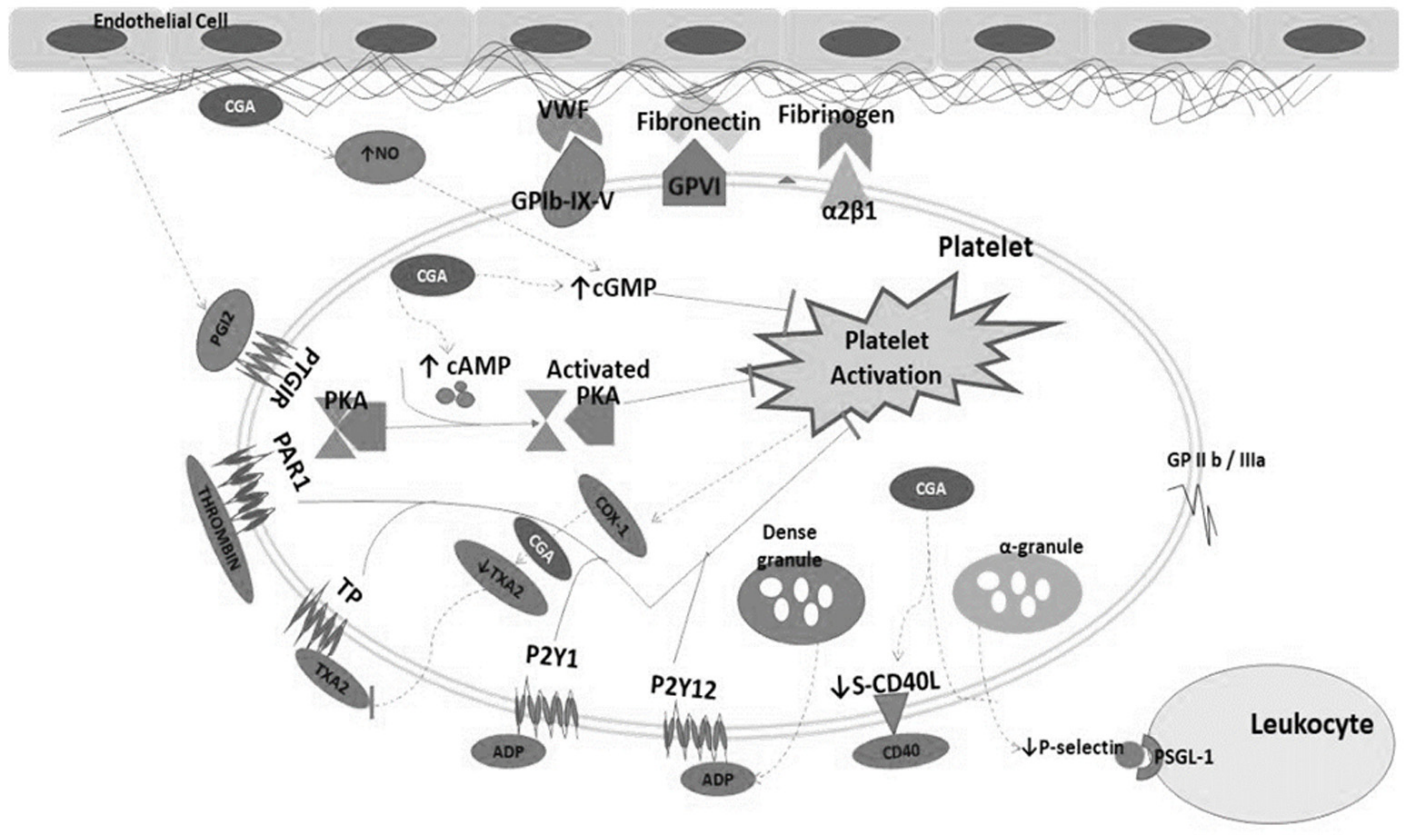
The mechanism of CGA in inhibiting platelet activation. NO, nitric oxide, vWF, von Willebrand factor; GPVI, glycoprotein VI; GPIb-IX-V, glycoprotein Ib-IX-V; PGI2, prostacyclin2, PTGIR, prostaglandin I2 receptor, PKA, protein kinase A, PAR1, protease activated receptor; TP, thromboxane A2 receptor; cAMP, cyclic adenosine monophosphate; cGMP, cyclic guanosine monophosphate, COX-1, cyclooxygenase-1; ADP, adenosine diphosphate; P2Y1, purinergic signaling receptor Y _1,; _P2Y12, purinergic signaling receptor Y _12_, GPIIb/IIIa, glycoprotein IIb/IIIa; S-CD40L, soluble cluster of differentiation 40 ligand; PSGL-1, P-selectin glycoprotein ligand-1.

CGA inhibits platelet activation by preventing their secretion and aggregation in a dose-dependent manner (0.1 to 1 mmol/L) ( Figure 1), by inhibiting ADP-dependent secretion and preventing their adhesion ^31^. CGA at these concentrations increases PKA activation or cAMP levels and decreases the inflammatory mediators of platelets (sP-selectin, CCL5, sCD40L, and IL-1β). Adenosine A _2A_ is the target of antiplatelet therapy, activation of this receptor results in an enhanced intracellular cAMP and the inhibition of platelet activation and aggregation. Molecular modelling has shown that CGA is compatible with adenosine A _2A_ receptor active site, which forms interactions with amino acids that specifically interact with A _2A_ ligands, such as NECA and adenosine. Interestingly, CGA has demonstrated a lower bleeding effect compared to that of aspirin ^31^.

CGA treatment has also been shown to inhibit TXA2 secretion and suppression of platelet aggregation. It is also an autacoidal molecule, a potent cyclooxygenase (COX)-1 inhibitor with cytochrome c reductase activity. Furthermore, CGA increases cAMP, cGMP, and intracellular Ca2R-antagonists formation ^31, 32^. These results suggest that CGA has antiplatelet activity through the increase of cAMP, cGMP and reduction of thromboxane A2 levels. Meanwhile, CGA shows an antiplatelet activity *in vitro* at a 50 nM concentration in mice. The same result was obtained *in vivo* after orally administering 400 mg per 30 g body weight to mice. In humans, this dose will be achieved after consuming about three cups of coffee rich in CGA, which will result in a low nM concentrations in the bloodstream ^33^.

## Mechanism of CGA in inhibiting hypertension

The evidence of CGA as a hypotensive agent has been suggested by many studies, for example in spontaneously hypertensive rats and mild essential hypertensive patients ^34^. CGA controls hypertension by reducing ROS through the attenuation of NAD(P)H-dependent superoxide. This effect inhibits the proliferation of smooth muscle cells *in vitro*, as well as *in vivo* by decreasing angiotensin-converting enzyme activity ^34^. Thus, CGA modulates the renin-angiotensin-aldosterone system. Ferulic acid, the CGA metabolite, has a considerable effect on blood pressure reduction. Its administration enhances acetylcholine-induced vasodilation and increases the bioavailability of NO in the arterial vasculature ^35^.

In addition, CGA extracted from green coffee was tested for its efficacy in lowering the blood pressure (BP) of hypertensive patients. A double-blind and randomized clinical trial on 117 subjects, where the intervention group received different quantities of the CGA extract for 28 days compared to a placebo group, showed that the extract markedly reduced BP without any adverse effects. Meanwhile, a meta-analysis showed that CGA reduced both systolic and diastolic BP ^36^.

Previous studies showed that CGA modulates NO levels in rat vessels ^37^, and therefore has a vasodilation effect. In addition, a study in humans investigated its acute effect on BP, NO status, and endothelial function; administration of 400 mg resulted in lower systolic and diastolic BP (−2.41 and −1.53 mmHg respectively; p < 0.05) compared to the control group. However, endothelial function and NO status were not significantly influenced. Ward *et al.* also investigated the acute effect of 900 mg of CGA on BP and endothelial function, and found that there was no marked effect on peak flow-mediated dilation. Meanwhile, there was continuous dilation improvement. Both 900 and 450 mg of CGA resulted in a high (p < 0.05) continuous flow-mediated dilation at 1 h, and higher at 4 h (0.44%) ^38^.

CGA has been shown to induce the production of NO and enhance antioxidant activity. For instance, caffeoylquinic consumption for eight weeks significantly enhanced NO production and reduced NADPH-dependent ROS in the aorta of hypertensive rats ^39^. Also, CGA has been shown to block the expression of the NADPH-oxidase gene, which helps to control vascular tone ^35^. These results indicate that CGA might induce NO production, decrease oxidative stress, and prevent some conditions, such as hypertension and vascular hypertrophy.

## Mechanism of CGA in inhibiting trans-endothelial migration

Atherosclerosis is a complex process that is initiated by inflammation and leukocyte migration to the inflamed area. Expression of cell adhesion molecules (CAM) on the endothelium and the attachment of monocytes to endothelium may play a major role in the early atherogenic process. During this process, adhesion molecules play a pivotal role in leukocyte cells recruitment and cellular matrix protein development. Ninjurin is a crucial molecule that increases the recruitment and activity of leukocytes during inflammation phase. A previous study showed the dose dependent manner inhibitory effect of CGA in mRNA Ninj1 gene expression that induced by LPS. Moreover, CGA significantly inhibited not only NO production but also the expression of COX-2 and iNOS, without any cytotoxicity. CGA also attenuated pro-inflammatory cytokines (including IL-1b and TNF-a) and other inflammation-related markers such as IL-6 in a dose-dependent manner ^40^. Moreover, CGA inhibited the nuclear translocation of NF-kB and blocked LPS-induced β2 integrin expression and L-selectin shedding. Meanwhile, it inhibited LECAM-1 expression on neutrophil membranes. CGA was also shown to inhibit immunoglobulin molecules by decreasing vascular CAM-1 expressions on the endothelium of human umbilical venule. However, another study suggested that its effects on the expression of PECAM-1 does not involve genetic synthesis ^25, 41^.

**Figure 2.**
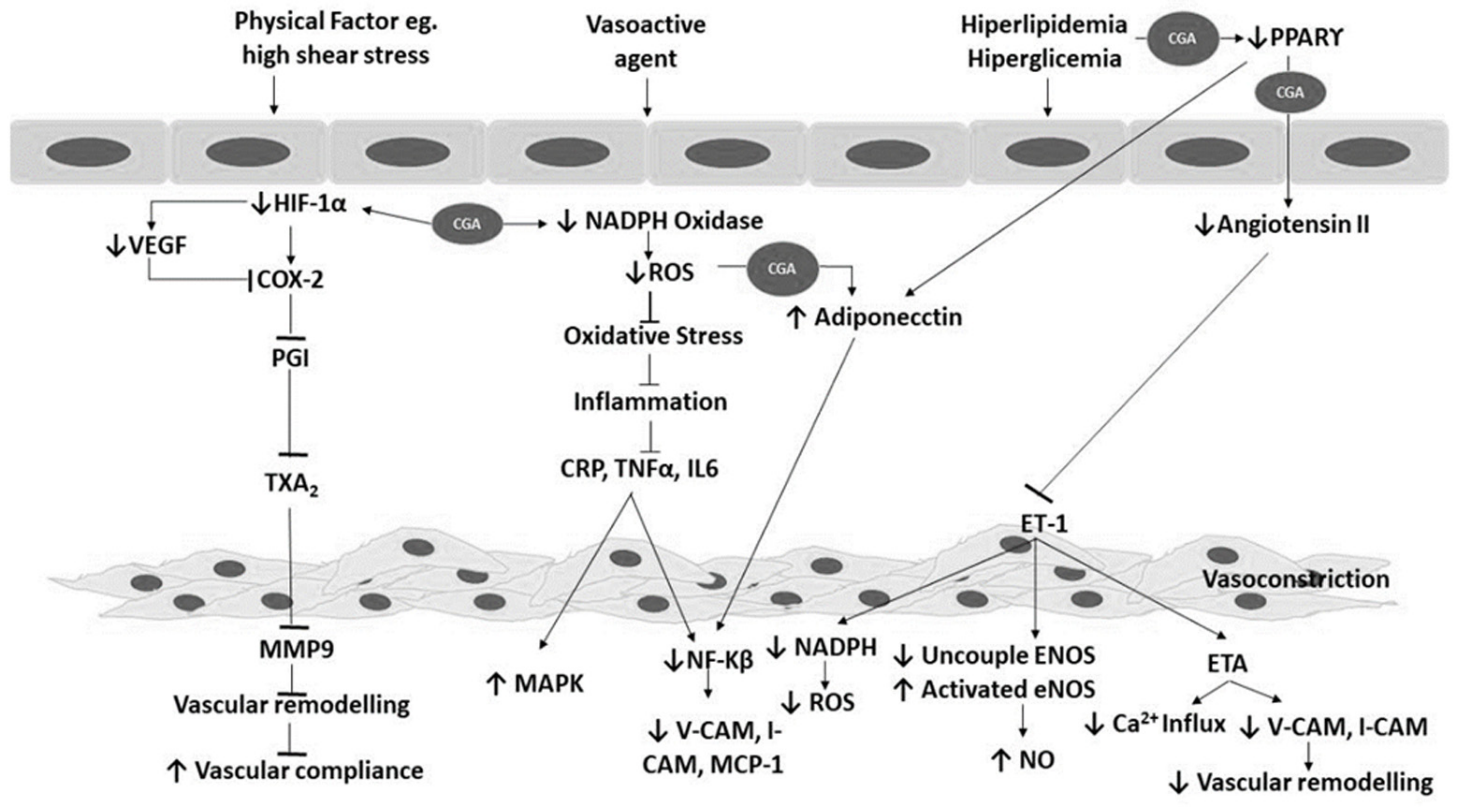
The mechanism of CGA in inhibiting atherosclerosis and hypertension. PPAR, peroxisome proliferator activated receptor; HIF-1α, hypoxia inducible factor1-α; VEGF, vascular endothelial growth factor; COX-2, cyclooxygenase-2; PGI, prostacyclin; TXA _2_, thromboxane A2; MMP9, matrix metalloproteinase 9; NADPH, Nicotinamide adenine dinucleotide phosphate; ROS, reactive oxygen species; CRP, C-reactive protein; TNFα, tumor necrosis factor α; IL6, interleukine 6; MAPK, mitogen-activated protein kinase; NF-kβ, nuclear factor kappa-B, V-CAM, vascular cell adhesion molecule-1, I-CAM, intercellular adhesion molecule; MCP-1, monocyte chemoattractant protein-1, ET-1, endothelin-1; ENOS, endothelial nitric oxidase; NO, nitric oxide, ETA, endothelin A.

A study by Chang *et al*. ^21^ showed that CGA treatment significantly reduced the concentration of proinflammatory cytokines that play an important role in the progression and development of atherosclerosis ( Figure 2). Its anti-inflammatory properties explained its inhibitory effects on CAM expression, as it suppressed ICAM-1, VCAM-1, and E-selectin expression, which is induced by IL-1β ^25^. However, it should be noted that consuming a high dose coffee might increase the concentration of homocysteine in human plasma that will consequently lead to ED ^42^. A previous study by Chang *et al.*, showed that CGA suppressed cytokine-induced CAM expression and inhibited p50 and p65 nuclear translocation in endothelial cells ^25^. Therefore, this study also showed that it reduced IL-1β-induced ROS production in human umbilical vein endothelial cells (HUVECs). Furthermore, CGA removed RO• and ROO•, as well as DPPH radicals, which are produced from LDL oxidation ^43– 45^. Finally, a previous study also showed that CGA at 50 and 25mmol/L inhibited U937 monocyte-like adhesion, expression of adhesion molecules, NF-KB translocation, and ROS production in HUVECs ^25^.

## Anti-angiogenic mechanism of CGA

Hypoxia-induced angiogenesis plays a pivotal role in the development of atherosclerotic lesions. It enhances endothelial cell and vascular smooth muscle cell proliferation through the HIF-1a–VEGF pathway, and contributes to vulnerable plaque progression leading to destabilization. During atherogenesis, the tunica intima is thickened due to cell and matrix accumulation, thus impairing oxygen diffusion. The microenvironment within the plaque is hypothesized to be an essential determinant of plaque progression. During hypoxic condition, several HIF-responsive genes are shown to be upregulated in atherosclerosis such as VEGF, endothelin-1, and matrix metalloproteinase-2 ^46^. Some studies suggest that CGA ameliorates hypoxia induced atherosclerosis via modulation of HIF-1α-VEGF pathway. A study in A549 cells, as well as in DU145 cells, showed that CGA treatment significantly decreased hypoxia-induced HIF-1α protein that consequently reduced the expression of VEGF. Moreover, during hyperglicemia CGA suppressed serum VEGF and HIF-1 alpha translocation. It was also suggested that CGA blocks *in vivo* and *in vitro* angiogenesis of HUVEC cells ^47^. In addition, CGA has been shown to phosphorylates VEGFR2, ERK 1/2 and AKT in order to inhibit VEGF-induced proliferation, migration, and invasion of HUVEC cells ^48^.

## CGA and vascular health in human studies

From various human studies ( Table 1), it can be seen that CGA administration in various doses resulted in favourable effect in improvement of cardiovascular function, through amelioration of flow mediated dilatation (FMD) after either acute or chronic administration of CGA. A single intake of CGA with the dose of 400 mg improved FMD and lowered BP. Moreover, it has been suggested that administration of low hydroxyhydroquinone CGA results in better improvement of FMD compared to that of high hydroxyhydroquinone ^36^.

**Table 1.** Effect of chlorogenic acid consumption on vascular health: evidence from human studies.

Design	Sample characteristics	Primary end points	Intervention	Results	References
Double-blind, randomized controlled crossover study	23 healthy men and women. All participants were regular tea (mean ± SD, 1.7 ± 1.5 cups/day) and coffee (mean ± SD, 1.7 ± 1.6 cups/day) consumers	Plasma RXNO, nitrite, and NOx Blood pressure FMD of the brachial artery	Single intake of 400 mg of chlorogenic acid (3-O- caffeoylquinic acid).	Lower mean SBP and DBP compared to those of control group. The markers of nitric oxide status and endothelial functions were not significantly affected.	36
Single-blind, randomized, placebo- controlled, crossover- within-subject	37 men and women with borderline or stage 1 hypertension	FMD	Single intake of beverage A that contained chlorogenic acids: 412 mg, hydroxyhydroquinone: 0.11 mg, and caffeine: 69 mg) or beverage B that contained chlorogenic acids: 373 mg, hydroxyhydroquinone: 0.76 mg, and caffeine: 75 mg	The intake of coffee with high chlorogenic acid and low hydroxyhydroquinone improved post pandrial FMD vasodilatation and reduced circulating 8-isoprostane levels	52
Single blind, randomized, controlled clinical trial	38 healthy men and 37 healthy women	Lipid profile and vascular function based on FMD, BP, NO metabolites.	8 week consumption of a medium CGA content (MCCGA; 420 mg) or high CGA content (HCCGA; 780 mg)	No significant differences in the lipid, FMD, BP, or NO plasma metabolite values were observed between the groups.	53
A double-blind, randomised, placebo controlled cross-over trial	17 healthy men and women. The participants consumed coffee regularly	FMD, BP, plasma nitrite concentrations	Single intake of 450 mg purified 5-CGA or 900 mg purified 5-CGA	No significant effect of 5-CGA, at 450 and 900 mg, on peak FMD response. However, there were significant improvements in mean post-ischaemic FMD response, particularly at the 1 h time point in this group of healthy individuals	38
Single-blind, randomized, controlled, crossover trial	20 healthy males	Reactive hyperemia ratio	CQA 140 mg/day for 4 months	Higher RHR compared to that of placebo group	54
Double-blind, placebo controlled, pilot study	16 healthy men	Cardio-ankle vascular index (CAVI), FMD, sympathetic nervous activity (SNA)	2 weeks consumption of a beverage contained 300 mg CGA	The CAVI change was significantly greater in the cGCE group than in the placebo group. In addition, FMD increased and SNA decreased in the cGCE group.	55
Double- blinded, randomized crossover trial	13 healthy men aged 30–60 years old	FMD	Single intake of a beverage contained 600 mg of CGA	The postprandial impairment of FMD was significantly improved compared to the placebo group.	56
Two randomized, controlled, crossover clinical trial	Study 1: 15 healthy males Study 2: 24 males	FMD	Study 1: single intake of a beverage containing 89 mg CGA or 310 mg CGA. Study 2: single intake of purified 5-CQA at a dose of 450 mg or 900 mg	CGA intake with low and high polyphenol acutely improved FMD	41
randomized acute clinical intervention study with crossover design	15 healthy men aged 20–60 years old	Reactive hyperemia index (RHI)	600 mg CQA	Higher RHI at 1.5 hours after ingestion significantly increased from the baseline value and was significantly different from that in the Glu group.	57
Randomized, placebo, controlled cross over design	7 healthy men and 5 healthy women	FMD	Ground caffeinated coffee contained 95 mg CGA and decaffeinated coffee contained 132 mg CGA	Higher FMD response in caffeinated coffee group	58
Single blind, randomized, placebo- controlled crossover trial	19 healthy males	FMD	Coffee polyphenol extract contained 355 mg CQA	Higher postpandrial FMD	24

FMD, flow mediated dilatation; RHI, reactive hyperemia index; CAVI, cardio-ankle vascular index; SNA, sympathetic nervous activity; NO, nitric oxide; RXNO, S-nitrosothiols and other nitrosylated species; NOx, nitric oxides comprising nitros(yl)ated species + nitrite; BP, blood pressure; SBP, systolic blood pressure; DBP, diastolic blood pressure; CQA, caffeoylquinic acid.

## Achieving high CGA benefit from coffee manufacturing process

Many procedures have been introduced in the coffee manufacturing process to achieve more benefits during coffee consumption. Previous studies have shown that roasting levels alter CGA content and antioxidant activity; lightly roasted coffee had more of this compound compared to other groups, and has a higher antioxidant activity based on 2,2-diphenyl-1-picrylhydrazyl (DPPH) assay ^44, 49^. The most abundant CGA isomer was 5-CQA with an estimate of 69–74% in the extracts, especially those from green beans ^50^. The 5-CQA content decreased to less than 85% in brews from non-roasted green beans obtained from the same location, and the total CGA content in the extracts of dark-, medium-, and light-roasted beans decreased to 80.60%, 62.91%, and 35.60% respectively ^51^. In addition, 4-CQA and 3-CQA were found at higher percentages in the light-roasted brew compared to green beans. Another study using ABTS and Folin-Ciocalteu assays showed that there is high antioxidant activity in medium and light-roasted brews ^50^.

High CGA also can be achieved through fermentation process. Some fermentation procedures had been proposed such as using Saccharomyces cereviciae and Bacillus subtilis strains. A fermentation procedure of coffee pulp using Saccharomyces cereviciae resulted in 400% richer CGA content. Interestingly, the addition of ultrasound treatment did not increase the yield from the extracted coffee pulp. Moreover, the use of Bacillus subtilis strains during fermentation process lead to 20% greater CGA content from green coffee bean extract ^59, 60^.

## Conclusion

CGA protects vascular health by inhibiting ED. Several mechanisms explain its effects on LPC injury and atherosclerosis, modulation of dual PPAR α/γ agonist, AMPK phosphorylation, adiponectin, and adiponectin receptors. It plays a role in reducing proinflammatory cytokine concentration that contribute to atherosclerosis development and progression. Furthermore, it suppresses the expression of E-selectin, VCAM-1, and ICAM-1, as well as decreases HOCl-induced oxidative damage in endothelial cells. In addition, CGA induces hemeoxygenase-1 and antiplatelet activity through thromboxane A2 (TXA2) reduction, and attenuates ROS by decreasing the production of NAD(P)H-dependent superoxide. Furthermore, it inhibits the activity of ACE and the proliferation of smooth muscle cells. Finally, it has been shown to block the HIF -1α/AKT signalling pathway, which plays a crucial role in the activation of VEGF and angiogenesis.

## Data availability

No data are associated with this article.
