# Supplementary material for: Cardiovascular protection effect of chlorogenic acid: focus on the molecular mechanism
Source: F1000Res. 2020 Dec 15;9:1462. [Version 1] doi: 10.12688/f1000research.26236.1 (PMC7927207; doi:10.12688/f1000research.26236.1)
Supplement: Peer review history

# Peer review: Reviewer response for version 1

**DOI:** 10.5256/f1000research.28955.r79914

**Published:** 2021-03-02

**Authors:** Suowen Xu

**Affiliations:** 1 Department of Endocrinology, University of Science and Technology of China (USTC), Hefei, 230037, China

This is a very comprehensive review of CGA in cardiovascular health and diseases. By reading through the whole manuscript, the message conveyed is scattered and not focused. For example, the authors can divide the Atherosclerosis related mechanisms into sub-sections: endothelial dysfunction, macrophage inflammation and foam cell formation, VSMC dysfunction, platelet activation etc. How other CVD protective actions, the authors can talk about cardiac hypertrophy and heart failure, as CVD is very broad, it is hard to give concise review and discussion on all forms of CVD. I would suggest to focus on hypertension and atherosclerosis in particular. The detailed molecular targets of CGA can be given in a pictorial way as new figures which is very important. 

 It is also good to include are there any clinical trials of CGA in Clinicaltrial.gov? What are unexplored and important in CGA research? In particular, CGA functions as more than antioxidant, which failed in most clinical trials.

Is the review written in accessible language?

Yes

Are all factual statements correct and adequately supported by citations?

Yes

Are the conclusions drawn appropriate in the context of the current research literature?

Yes

Is the topic of the review discussed comprehensively in the context of the current literature?

Yes

Reviewer Expertise:

natural product pharmacology, atherosclerosis, endothelial cell biology

I confirm that I have read this submission and believe that I have an appropriate level of expertise to confirm that it is of an acceptable scientific standard, however I have significant reservations, as outlined above.

# Peer review: Reviewer response for version 1

**DOI:** 10.5256/f1000research.28955.r77826

**Published:** 2021-02-24

**Authors:** Arrigo F.G. Cicero

**Affiliations:** 1 Hypertension Research Unit, University of Bologna, Bologna, Italy

I’ve read with attention the narrative review by Lukitasari et al. on the pharmacological activities of chlorogenic acid on vascular health. The review is interesting, well-organized, overall well-written and updated. My only suggestion is to report quantitative results when speaking about human studies. The conclusion should also include 1-2 sentences on the perspective of research in this field.

Is the review written in accessible language?

Yes

Are all factual statements correct and adequately supported by citations?

Yes

Are the conclusions drawn appropriate in the context of the current research literature?

Yes

Is the topic of the review discussed comprehensively in the context of the current literature?

Yes

Reviewer Expertise:

Cardiovascular disease prevention in clinical settings

I confirm that I have read this submission and believe that I have an appropriate level of expertise to confirm that it is of an acceptable scientific standard.

# Peer review: Reviewer response for version 1

**DOI:** 10.5256/f1000research.28955.r76178

**Published:** 2021-02-01

**Authors:** Katalina Muñoz-Durango

**Affiliations:** 1 Nutresa Business Group, Vidarium - Nutrition, Health and Wellness Research Center, Medellín, Colombia

I consider that the topic is of great interest. Chlorogenic acids are important bioactive compounds that have been a matter of extensive research in the last decades. Therefore, a review related to the cardiovascular protective effect of chlorogenic acid: focus on the molecular mechanism, is convenient and necessary. Nevertheless, some important aspects should take into account.

 INTRODUCTION

- Instead of "Chlorogenic acid (CGA) is the primary polyphenol" I suggest  "Chlorogenic acid (CGA) is the primary phenolic compound"
- Change "compound of phenol" by "phenolic compound"
- Please, verify the sentence “Chlorogenic acid (CGA) is a compound of phenol that consists of a caffeic and quinic acid moiety; therefore, it is also called 5-O-caffeoylquinic acid (5-CQA), although many authors refer to it as 3-CQA”  

It is well known that the major CGAs isomers in coffee include chlorogenic (3-CQA), cryptochlorogenic (4-CQA) and neochlorogenic (5-CQA) acids, as well the dimers 3,4-diCQA, 3,5-diCQA and 4,5-diCQA [1,2].

- Add the reference: A cup of coffee (200 ml) consists of 20–350 mg CGA, which contains 35–175 mg of caffeic acid.
- Ref 3 is used to indicate the average of CGA consumed by coffee drinkers, please do not use a reference related to green coffee extract, and be sure that you are talking about chlorogenic acid and not chlorogenic acids (CGAs), that is, in my opinion, the right on in this case. Authors should clarify when they refer to chlorogenic acid as the chemical entity (the ester of caffeic acid and (−)-quinic acid) or when they want to talk about chlorogenic acids (CGAs) as the group of phenolic compounds formed by a hydroxycinnamic acid and quinic acid classified by the number, position and type of hydroxycinnamic acid. The most common groups of CGAs are *ρ*-coumaroylquinic acids, feruloylquinic acids, caffeoylquinic acids (CQAs) and dicaffeoylquinic acids (diCQAs). As I mentioned before, to date, the major CGAs isomers in coffee include chlorogenic (3-CQA), cryptochlorogenic (4-CQA) and neochlorogenic (5-CQA) acids, as well the dimers 3,4-diCQA, 3,5-diCQA and 4,5-diCQA.

ENDOTHELIAL DYSFUNCTION (ED)

- Taking into account that the paper is related to molecular mechanism, I suggest the author consider go further back “ED is a cardiovascular events predictor and considered as the initial stage of atherosclerosis development”. Please, consider in this section to mention the key atherogenic process related to ED. Macrophage foam cells and their role in atherosclerosis. Oxygen species (ROS) production, inflammatory responses and accumulation of lipids, which lead to fatty streak formation in the vascular wall. The massive uptake of oxLDL by macrophages via scavenger receptors (SR-A and CD36) and lectin-like oxLDL receptor-1 (LOX-1), etc. Oxylipins and prostaglandins, among others.

CHLOROGENIC ACID

- This section is extremely general, in my opinion, it could be added in any other section.
- Idem previous sections. Please be clear with the chemistry, avoid sentences like “It is also called 5-O-caffeoylquinic acid (5-CQA), although some authors refer to it as 3-CQA”, it is not important if other authors confuse the nomenclature, do it right is enough, in my opinion. But, it is a matter of discussion in this paper, please reference the wrong ones, and discuss. Please, check: “A cup of coffee (200 ml) consists of 20–350 mg CGA” You should make the difference between the chemical compound (CGA) and the group of the isomers named CGAS (I already mentioned it before).

MECHANISM OF CGA IN INHIBITING ATHEROSCLEROSIS

- Add reference: CGA has been shown to significantly increase mRNA levels of PPARγ, LXRα, ABCA1 and ABCG1, as well as the transcriptional activity of PPARγ.
- The next section is not adequately referenced. The only bibliography added does not correspond to the effect of chlorogenic acids.

“CGA has been shown to significantly increase mRNA levels of PPARγ, LXRα, ABCA1 and ABCG1, as well as the transcriptional activity of PPARγ. In addition, a cholesterol efflux assay showed that three major metabolites, caffeic, ferulic and gallic acids, significantly stimulated cholesterol efflux from RAW264.7 cells. These results suggest that CGA potently reduces atherosclerosis development in ApoE ^−/−^ mice and promotes cholesterol efflux from RAW264.7 macrophages  (14)”

- Related to CGAs in inhibiting atherosclerosis, authors should consider literature that mentioned their in vitro and in vivo (clinical studies) effects on oxylipins, isoprostanes, and prostaglandins.

MECHANISM OF CGA IN INHIBITING HYPERTENSION

 I suggest the authors consider the expression “inhibiting hypertension”, for this section. In my opinion, it exceeds what the studies have found. There are conflicting results related to this topic.

- Ref 38: important to mention that There was no significant effect of any of the treatments on BP.
- In the last paragraph on the action add caffeoylquinic “acid”

In this section, the authors analyzed FMD, BP, and NO, nevertheless, in the section “CGA and vascular health in human studies”, they go back to the same topics. I would like to see more linkage among the topics, and more discussion related to table 1. In this table, you include 11 references, but in the section authors only discuses ref 36.

 ACHIEVING HIGH CGA BENEFIT FROM COFFEE MANUFACTURING PROCESS

 This section is extremely general. I don't see the point to have an independent section. This information could be added to the introduction, in a general manner. The paper is not about coffee, is about CGAs. Coffee is a very important source, but the focus of the paper is more oriented to the molecule 5-CQA, and sometimes to the group of isomers (CGAs). Authors must clarify it, as well as when use references related to coffee.

 CONCLUSION

 The conclusion leaves out many of the aspects mentioned and discussed.

Is the review written in accessible language?

Yes

Are all factual statements correct and adequately supported by citations?

Partly

Are the conclusions drawn appropriate in the context of the current research literature?

Partly

Is the topic of the review discussed comprehensively in the context of the current literature?

Partly

Reviewer Expertise:

Antioxidants, mass spectrometry, biomarkers, bioavailability, clinical studies, obesity, cardiovascular health, chronic non-communicable diseases

I confirm that I have read this submission and believe that I have an appropriate level of expertise to confirm that it is of an acceptable scientific standard, however I have significant reservations, as outlined above.

## References

[1] : Chlorogenic acid compounds from coffee are differentially absorbed and metabolized in humans. *J Nutr*.2007;137(10) : 10.1093/jn/137.10.2196 2196-201 10.1093/jn/137.10.2196 17884997

[2] : Phytochemicals in Coffee and the Bioavailability of Chlorogenic Acids.2011; 10.1002/9781444347098.ch5 143-168 10.1002/9781444347098.ch5
